# Evaluation of Postbiotics from *Lactiplantibacillus plantarum* and *Bacillus mojavensis* Fermented in Aguamiel on Their Cytotoxic Effect in Breast Cancer Cells, Their In Vivo Effects, and Processing by Spray Drying

**DOI:** 10.3390/nu18172831

**Published:** 2026-08-28

**Authors:** Adelfo García-Ceja, Karen Tatiana Zuluaga-López, Rosa Isela Ortiz-Basurto, Daniel Tapia-Maruri, María de Lourdes Meza-Jiménez, Gabriel Abraham Cardoso-Ugarte, M. Jiménez-Fernández, Porfirio Nava, Beatriz Pérez-Armendáriz

**Affiliations:** 1 Vicerrectoría de Docencia, Facultad de Biotecnología, Universidad Popular Autónoma del Estado de Puebla, 21 Sur No. 1103, Barrio Santiago, Puebla 72410, Mexico; adelfo.garcia@upaep.edu.mx (A.G.-C.); karentatiana.zuluaga@upaep.edu.mx (K.T.Z.-L.); 2 Ingeniería en Industrias Alimentarias, TecNM-Instituto Tecnológico Superior de Venustiano Carranza, Av. Tecnológico S/N, Ciudad Lázaro Cárdenas, Puebla 73049, Mexico; 3 Laboratorio Integral de Investigación en Alimentos, TecNM-Instituto Tecnológico de Tepic, Tepic 63175, Mexico; riobasurt@ittepic.edu.mx; 4 Centro de Desarrollo de Productos Bióticos, Instituto Politécnico Nacional, Yautepec 62739, Mexico; dmaruri@ipn.mx; 5 Vicerrectoría de Ciencias de la Salud, Facultad de Nutrición, Universidad Popular Autónoma del Estado de Puebla, 21 Sur No. 1103, Puebla 72410, Mexico; marialourdes.meza@upaep.mx; 6 Vicerrectoría de Docencia, Escuela de Gastronomía, Universidad Popular Autónoma del Estado de Puebla, 21 Sur No. 1103, Puebla 72410, Mexico; gabrielabraham.cardoso@upaep.mx; 7 Centro de Investigación y Desarrollo en Alimentos, Universidad Veracruzana, Xalapa 91000, Mexico; maribjimenez@uv.mx; 8 Department of Physiology, Biophysics, and Neurosciences, Center for Research and Advanced Studies of the Polytechnic Institute, Mexico City 07360, Mexico; pnava@fisio.cinvestav.mx

**Keywords:** postbiotics, microencapsulation, spray drying, cell line assays, functional food ingredients

## Abstract

Background/Objectives: Postbiotics derived from beneficial bacteria have emerged as promising bioactive ingredients owing to their enhanced stability, safety, and functional versatility relative to live microorganisms. Methods: The present study was structured in two complementary stages: (i) biological characterization of liquid microfiltrates obtained from *Lactiplantibacillus plantarum* and *Bacillus mojavensis* fermented in aguamiel, and (ii) technological stabilization of these postbiotics through microencapsulation by spray drying, with a view to their potential incorporation into food matrices. Results&Conclusions: In the first stage, biological activity was assessed in vitro using MDA-MB-231 human breast cancer cells and in vivo in C57BL/6J mice, in order to evaluate safety and gastrointestinal effects. Both postbiotics significantly reduced cell viability relative to controls, consistent with antiproliferative activity. In vivo, no macroscopic intestinal damage was observed, supporting their safety profile. Histomorphometric analysis revealed preserved intestinal architecture, with elongation of villi and crypts and increased mucin 2 production, indicating strengthened mucosal barrier integrity; these effects were more pronounced in the *Bacillus mojavensis*-treated group. In the second stage, the spray-dried powders exhibited adequate physicochemical stability, supporting their potential application as functional food ingredients. The spray-dried powders exhibited adequate physicochemical stability, supporting their potential application as functional food ingredients.

## 1. Introduction

The Food and Agriculture Organization of the United Nations defines functional foods as those that, beyond providing basic nutrition, contain bioactive components capable of exerting beneficial health effects and contributing to the prevention of chronic disease [1]. Within this category, products incorporating probiotics, prebiotics, postbiotics, and synbiotics have gained particular relevance owing to their capacity to modulate the composition, diversity, and metabolic activity of the intestinal microbiome [2]. Probiotic bacteria, particularly those belonging to the genera *Lactobacillus* and *Bifidobacterium,* are widely used in fermented dairy products [3,4]. To exert beneficial effects on the host, they must be administered at adequate concentrations (≥10^6^ CFU/g or mL), with an estimated intake of 10^8^–10^9^ CFU/day [3]. However, maintaining cell viability remains one of the main technological challenges, since factors such as acidic pH, water activity, oxygen exposure, and processing conditions can compromise bacterial stability and functionality [5,6]. Prebiotics such as inulin and fructooligosaccharides (FOS), in turn, act as selective substrates that stimulate the growth of beneficial intestinal bacteria. Non-conventional sources such as aguamiel from pulque agave have attracted interest due to their high content of fermentable sugars, inulin, vitamins, and minerals, which confer considerable biotechnological potential as a natural substrate for fermentative processes [6,7].

Interest in postbiotics has grown markedly in recent years. According to the International Scientific Association for Probiotics and Prebiotics (ISAPP), postbiotics are defined as preparations of inanimate microorganisms and/or their structural components that confer a health benefit on the host [8]. This consensus definition characterizes a postbiotic on the basis of four criteria: (i) a properly identified and characterized progenitor microorganism; (ii) a clearly delineated production/inactivation process; (iii) a demonstrated health benefit in the host; and (iv) safety for the intended use [9,10]. Because postbiotics do not require cell viability to exert biological activity, they offer important technological advantages, including greater stability during processing and storage [2]. Postbiotics encompass a wide range of bioactive molecules, including organic acids, short-chain fatty acids, antimicrobial peptides, bacteriocins, exopolysaccharides, enzymes, and cell-wall fragments [9]. Their production typically involves controlled microbial inactivation by thermal or non-thermal technologies, followed by recovery of cellular fractions and/or supernatants retaining biological activity [2]. Throughout the present study, the term postbiotic is used in accordance with this consensus definition, and all four criteria described above are addressed.

From a molecular standpoint, the mechanisms of action proposed for postbiotics include: (i) modulation of the immune system through activation of receptors such as Toll-like receptors (TLRs) and nucleotide-binding oligomerization domain-containing proteins (NODs); (ii) reinforcement of the intestinal barrier via induction of tight-junction proteins; (iii) antimicrobial activity associated with the production of organic acids and bioactive peptides; and (iv) interference with quorum-sensing mechanisms related to microbial virulence [11,12,13]. Postbiotics can further exert beneficial effects even in the absence of viable microbiota, broadening their potential application across clinical and food contexts [9]. In food technology, their inactivated nature facilitates incorporation into matrices in which live probiotics are constrained, such as products subjected to thermal treatment or prolonged storage [8]. Accordingly, microencapsulation and spray drying have become established strategies for stabilizing bioactive compounds, protecting them against oxidation, moisture, and extreme pH, and enabling their formulation as powders [14,15,16].

In the present study, the biological activity and technological feasibility of postbiotic preparations obtained by aguamiel fermentation with *Lactiplantibacillus plantarum* and *Bacillus mojavensis* BF2a1 were evaluated, using three independent preparation processes. First, cell-free postbiotics (MRS-fermented culture, centrifuged and filtered through a 0.22 µm membrane) were analyzed for their effect on the viability of MDA-MB-231 breast cancer cells in an in vitro model. Second, heat-inactivated postbiotics (aguamiel-fermented culture used as the administration vehicle, centrifuged and heat-inactivated without membrane filtration owing to filter clogging) were evaluated for their impact on intestinal tissue in an in vivo murine model. Third, and independently of the biological assays, aguamiel-fermented microfiltrates were spray-dried to obtain a powdered formulation, whose physicochemical, structural, and thermal properties were characterized with the aim of developing a postbiotic-based functional food powder (PBFF) for potential application in food or nutraceutical matrices. Selection of the two strains and the fermentation substrate was grounded in the existing literature: *L. plantarum* was chosen for its well-documented immunomodulatory and probiotic properties [17], and *B. mojavensis* for its demonstrated probiotic potential and regional relevance as a strain native to aguamiel [18]. Aguamiel was selected as the fermentation substrate because it constitutes the natural ecological niche of both strains, providing a biologically coherent rationale for their combined use in this study. This work therefore aimed to integrate initial biological validation of a postbiotic with the development of a technological strategy for obtaining a functional ingredient intended for incorporation into food and nutraceutical matrices.

## 2. Materials and Methods

### 2.1. Bacterial Strains and Substrate

The *Lactiplantibacillus plantarum* and *Bacillus mojavensis* BF2A1 strains used in this study were obtained from the culture collection of the Biotechnology Laboratory at UPAEP. Both strains were cultured on MRS agar and in MRS broth (Difco™ BD, Sparks, MD, USA) at 37 °C for 24 h and 48 h, respectively. Aguamiel was obtained from the Tinacal “Los Tuzos,” located in Singuilucan, Hidalgo, Mexico. For conditioning, aguamiel was diluted 1:1 with purified water (Epura^®^, GEPP, Puebla, Mexico), adjusted to 13 °Brix with sucrose, and subsequently pasteurized at 80 °C for 30 min [6]. For growth in aguamiel, the substrate was inoculated with pre-inocula of *L. plantarum* and *B. mojavensis* and fermented at 36 °C for 48 h.

### 2.2. Preparation of Postbiotics

Three independent preparation processes were used, according to the intended application: in vitro cell-line assays, in vivo murine assays, and powder formulation. For the in vitro assays, the MRS-fermented culture was centrifuged (4000 rpm, 10 min) to separate the cell biomass from the supernatant, which was subsequently filtered through a 0.22 µm membrane (syringe filter) to obtain the postbiotic.

For the in vivo murine assays, the aguamiel-fermented culture (used as the administration vehicle) was centrifuged (4000 rpm, 10 min) and directly heat-inactivated at 96 °C for 5 min, without membrane filtration, as this step caused clogging of the filter owing to the composition of the aguamiel matrix.

To confirm the efficacy of heat inactivation in both biological-assay preparations, a sterility test was performed independently for each matrix. Positive controls consisted of a sample of the raw fermented culture (for both matrices) and, for the MRS-derived preparation only, an additional sample taken after centrifugation and 0.22 µm filtration but prior to heat inactivation. All samples, together with the corresponding heat-inactivated preparations, were plated in parallel: 200 µL of each sample was spread onto MRS agar and incubated at 37 °C for 48 h, with an intermediate check at 24 h. All positive controls showed abundant microbial growth with the expected colonial morphology, confirming a viable microbial load prior to heat treatment in both matrices. In contrast, no microbial growth was observed on plates corresponding to either heat-inactivated preparation, confirming that heat treatment effectively eliminated viable vegetative cells and spores, including those of *B. mojavensis*, in both the MRS- and aguamiel-derived postbiotic preparations.

For the powdered formulation, microfiltration was performed using the pilot-scale system designed by Miramontes-Escobar et al. (2024) [19], with modifications; no ultrasonic probe or ultrasonic treatment was applied during microfiltration. The pilot system consisted of a tubular TiO_2_ ceramic membrane (Tami Industries^®^, Nyons, France) measuring 58 cm in length and 2.54 cm in diameter, with 23 multichannels (0.35 cm hydraulic diameter), a pore size of 0.22 µm, and a filtration area of 0.35 m^2^. The system was operated at a pump frequency of 40 kHz, a cross-flow velocity of 6 m·s^−1^, a transmembrane pressure of 1.65 bar, and a temperature of 30 °C. The resulting postbiotic was subsequently spray-dried, as described in Section 2.3.1, and was not subjected to heat inactivation or used in any biological assay.

### 2.3. Phase 1: Cellular and Murine Models

#### 2.3.1. Cell Line

The MDA-MB-231 human breast cancer cell line (ATCC, Manassas, VA, USA; Cat. No. HTB-26) was used. Cells were cultured in supplemented RPMI 1640 medium (Gibco™, Thermo Fisher Scientific, Waltham, MA, USA; RPMI + 10% fetal bovine serum + antibiotics), seeded, and incubated (37 °C, 5% CO_2_, 48 h) until the required confluence was reached. Cells were then treated in triplicate with each postbiotic at serial concentrations of 30%, 15%, and 7.5% (*v*/*v*), calculated relative to the final volume of complete cell culture medium. An MRS broth control (subjected to the same centrifugation and heat-inactivation process, without bacterial inoculation) was included at equivalent concentrations to rule out possible cytotoxic effects arising from the culture medium itself, independent of the postbiotics’ bioactive metabolites. DMSO (10%) served as a positive control for cytotoxicity [20], a concentration documented to reliably induce cytotoxic effects in cancer cell lines, including breast cancer cells [21]. Treated cells were incubated for a further 24 h (37 °C, 5% CO_2_), after which the MTT assay was performed using 3-(4,5-dimethylthiazol-2-yl)-2,5-diphenyltetrazolium bromide (MTT; Sigma-Aldrich, Merck KGaA, Darmstadt, Germany; Cat. No. M2128) following the standard protocol.

#### 2.3.2. Animals

Female C57BL/6J mice aged 6–8 weeks, obtained from the animal facility of the Centro de Investigación y de Estudios Avanzados del Instituto Politécnico Nacional (CINVESTAV-IPN), were used. Animals were housed in groups of three in transparent polycarbonate cages under standard environmental conditions (controlled light/dark cycle, temperature, and humidity), with free access to standard laboratory rodent chow and water *ad libitum*, except during administration of experimental treatments in the drinking water. Animals were assigned to four treatment groups (*n* = 3 per group; total *n* = 12) by simple randomization per cage: water (control), aguamiel, *L. plantarum* postbiotic, and *B. mojavensis* postbiotic. Conditioned aguamiel served as the vehicle for postbiotic administration.

Following acclimatization, treatments were administered by adding aguamiel or the corresponding postbiotic to the drinking water at 5% (*v*/*v*), provided *ad libitum*; water was renewed every three days, and consumption was recorded daily per group, while body weight was assessed individually and daily. No formal predefined exclusion criterion was established; however, no animal was excluded from the analysis, as all remained clinically healthy throughout the experiment. On day eight, a final assessment was performed prior to euthanasia by cervical dislocation, after which the small and large intestines were dissected, cleaned, and stored in histological cassettes Tissue-Tek® cassettes (Sakura Finetek USA, Inc., Torrance, CA, USA) for subsequent paraffin embedding, sectioning, and processing.

##### Ethical Considerations

All animal procedures were reviewed and approved by the Institutional Committee for the Care and Use of Laboratory Animals (CICUAL) of CINVESTAV-IPN (approval no. 025-23, project identification 0186-16, approved on 22 August 2023, valid through 31 December 2025), in accordance with the Mexican Official Standard NOM-062-ZOO-1999 [22] for the care and use of laboratory animals. The sample size was not determined by an a priori power calculation; group sizes were instead defined based on resource optimization and the 3Rs principles (Replacement, Reduction, and Refinement), consistent with precedents established in the relevant literature. Euthanasia was performed individually by trained personnel via cervical dislocation, a swift technique that ensured immediate loss of consciousness and death (confirmed by absence of heartbeat), thereby minimizing animal distress.

#### 2.3.3. Tissue Processing and Immunohistochemistry

Lysozyme 1 (anti-Lysozyme 1; Abcam, Cambridge, MA, USA) and Mucin 2 (anti-MUC2; Santa Cruz Biotechnology, Dallas, TX, USA), were analyzed by immunohistochemistry in 5 µm intestinal histological sections. Tissue was fixed in 10% formalin prior to paraffin embedding, sectioning, and staining, all performed by a specialized external histology service. Representative images were captured with an optical microscope (1.5×; 5.82 µm/px). For quantitative analysis, labeled cells were counted in five random fields per tissue section (14.0×; 0.73 µm/px). Cell counts and intestinal tissue (colon) length measurements were performed using ImageJ version 1.54 (National Institutes of Health, Bethesda, MD, USA).The length of intestinal crypts in the small intestine and colon was determined by selecting the ten longest and most complete villi/crypts per treatment per mouse, following the method described by Adji et al. (2019) [23]. Measurements were performed by a single evaluator who was aware of the experimental group identity of each sample.

### 2.4. Phase 2: Microencapsulation and Characterization

#### 2.4.1. Preparation of Suspensions

Microfiltrates obtained from fermented aguamiel were mixed with wall materials at a 1:4 ratio (microfiltrate: wall material) until a total solids content of 30% was reached. Mixtures were homogenized at 20,000 rpm for 5 min. The combinations of microfiltrates and wall materials are shown in Table 1. Encapsulating materials were as follows: fructans (F) from *Agave tequilana* Weber var. azul, supplied by Campo Azul (Guadalajara, Mexico); gum arabic (GA), from Diquítra (Jalisco, Mexico); whey protein isolate (WPI), from Hilmar Ingredients (Dalhart, TX, USA); maltodextrin (DE 10; MD), from Ingredion (Jalisco, Mexico); and commercial sucrose, from Zulka (ZUCARMEX, Sinaloa, Mexico).

#### 2.4.2. Microencapsulation of Postbiotics

Microencapsulation was carried out using a pilot-scale spray dryer (Cima Industries Inc., LPG-5, Changzhou, China) equipped with a 50 mm rotary atomizing disk, under previously established operating conditions: inlet air temperature of 160 °C, outlet temperature of 80–90 °C, feed flow rate of 1.66 × 10^−4^ L·s^−1^, and air pressure of 0.4 MPa to drive the rotary atomizer [14,24].

#### 2.4.3. Physicochemical Characterization of Powders

Moisture content was determined using the AOAC (2007) [25] method. Water activity (a_w_) was measured using an Aqualab 4TEV meter (Pullman, WA, USA) at 25 °C. pH measurement was performed according to Hernández-López et al. (2018) [26]. Color parameters L*, a*, b*, and hue angle (H°) were determined using a colorimeter (Chroma Meter CR-301, Minolta Co., Osaka, Japan).

#### 2.4.4. Evaluation of Physical Properties of Powders

Bulk density (ρb) and tapped density (ρt) were determined according to Juárez-Trujillo et al. (2024) [15]. Hygroscopicity was measured following Hernández-López et al. (2018) [26]. Process yield, compressibility index (CI), and Hausner ratio (HR) were calculated according to Hernández-Nava et al. (2020) [27]. Solubility and wettability were determined according to Juárez-Trujillo et al. (2024) [15].

#### 2.4.5. Powder Morphology

##### Scanning Electron Microscopy (SEM)

The morphology of microencapsulated powders from aguamiel fermented with *L. plantarum* and *B. mojavensis* was analyzed with a JEOL JSM-7600F scanning electron microscope (Tokyo, Japan) at 1000× magnification for each sample.

##### Confocal Laser Scanning Microscopy (CLSM)

Samples were analyzed by confocal microscopy (CLSM, Zeiss LSM800, Jena, Germany) coupled to an AxioCam HD color camera (Carl Zeiss, Model 305, Germany) using ZEN 2.6 software [28].

##### Particle Size Determination

Particle size distribution of the powders was determined using a dynamic light scattering particle analyzer Nanotrac Wave II, Microtrac Inc. (York, PA, USA). Dispersion (Span) was calculated following Nordin et al. [29], where *D*_90_, *D*_10_, and *D*_50_ represent the particle diameters at the 90, 10, and 50 percentiles of the cumulative distribution, respectively.

(1) $$Span=\frac{\left(D_{90}-D_{10}\right)}{D_{50}}$$

##### Glass Transition Temperature (Tg)

Tg was determined by differential scanning calorimetry (DSC) using a Perkin Elmer DSC7 (Boston, MA, USA) equipped with a cooling system. Approximately 10 mg of each powder sample was weighed into aluminum DSC pans under continuous nitrogen purge (100 mL/min). Samples were heated from 0 to 250 °C at 5 °C/min to simulate spray-drying processing conditions.

##### Fourier Transform Infrared Spectroscopy (FT-IR)

Functional groups were analyzed by FT-IR spectroscopy (VERTEX 70, Bruker (Billerica, MA, USA) with a diamond attenuated total reflectance (ATR) cell over a spectral range of 4000–500 cm^−1^, at a resolution of 4 cm^−1^ and 64 scans per sample [28].

### 2.5. Statistical Analysis

Results were analyzed using a completely randomized design with three replicates per treatment and are expressed as mean ± standard deviation. Data were organized, and absorbance values converted to percentages, in Excel (Microsoft 365). Statistical analysis was performed by one-way ANOVA (*p* < 0.05); when significant differences were detected, Tukey’s post hoc multiple comparison test was applied using GraphPad Prism 9 software (Dotmatics, San Diego, CA, USA).

## 3. Results

### 3.1. Postbiotics on Cell Lines

**Figure 1:**
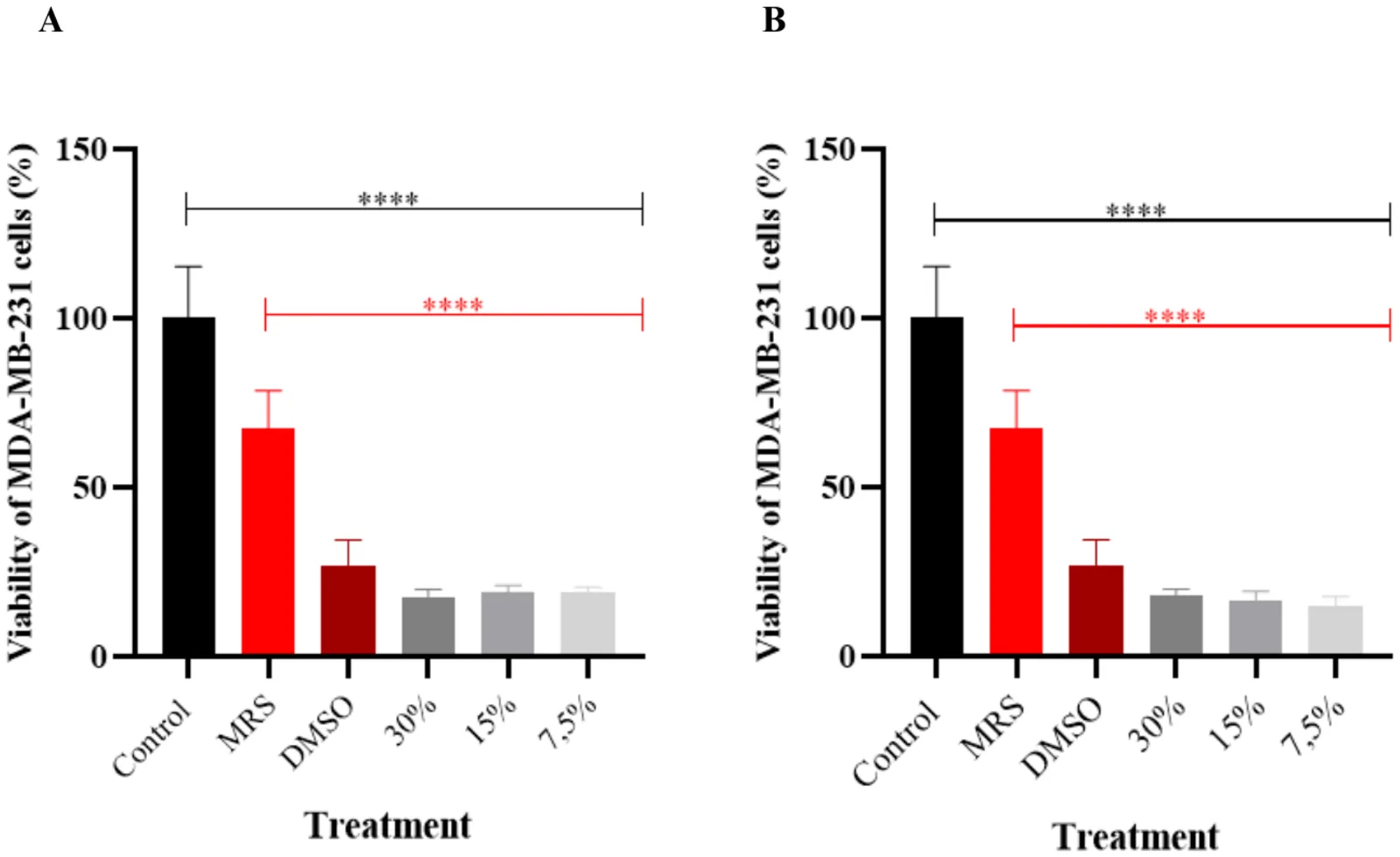
Effect of postbiotics on the viability of MDA-MB-231 cells (**A**) *L. plantarum* postbiotics. (**B**) *B. mojavensis* postbiotics. DMSO (10%) was used as an independent cytotoxicity reference control. Data represent the mean ± SD of three independent experiments performed in technical triplicate. (**** *p* < 0.0001).

Postbiotics from *L. plantarum* and *B. mojavensis* reduced the viability of MDA-MB-231 breast cancer cells, with no statistically significant differences among the concentrations evaluated (Figure 1). Significant differences (*p* < 0.0001), however, were found between the RPMI and MRS controls and the postbiotic treatments and the positive control (DMSO).

### 3.2. Effect of Postbiotics on Murine Intestinal Tissue

**Figure 2:**
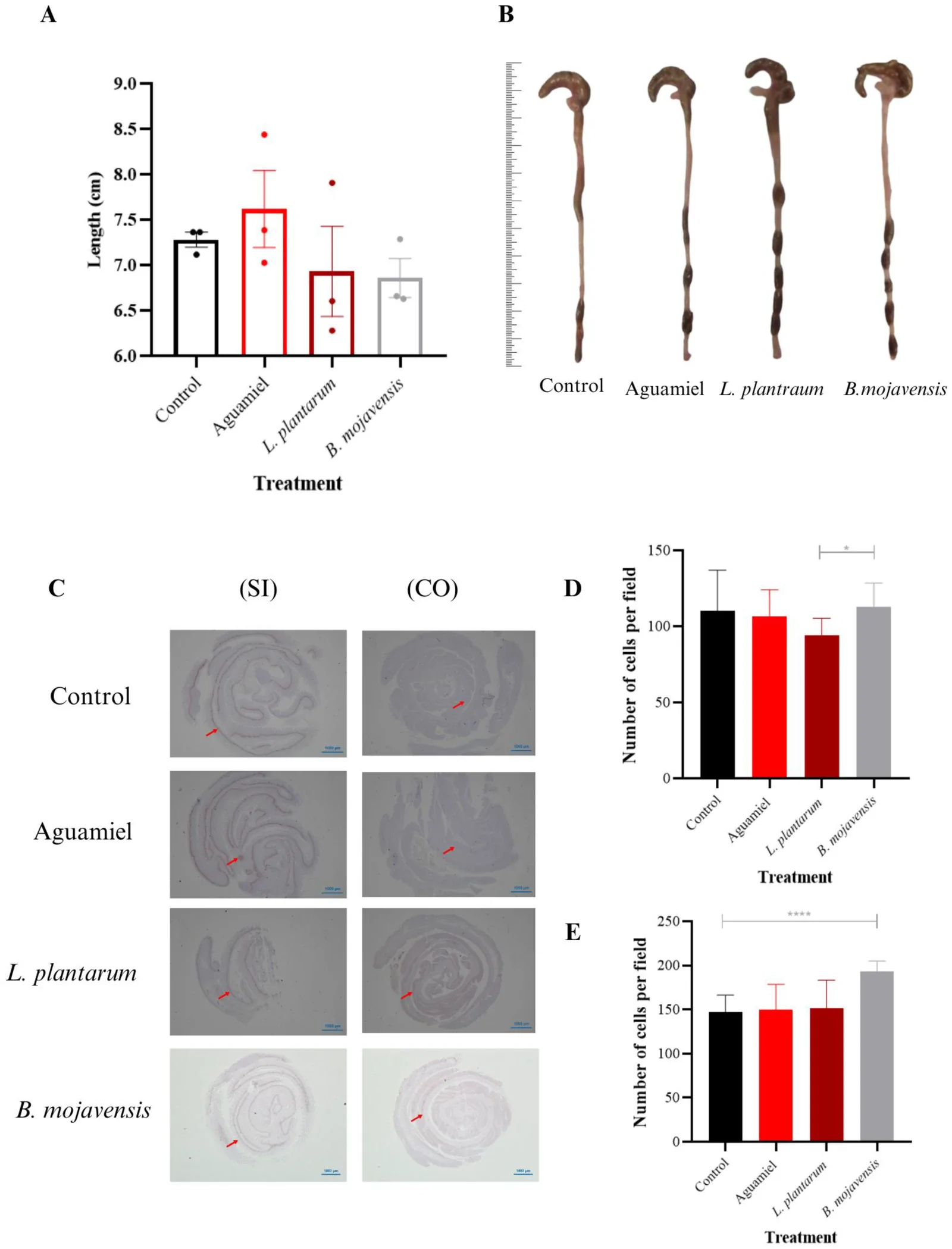
Effects of treatments on large intestine morphology and intestinal immunohistochemical markers in C57BL/6J mice. (**A**) Length of the large intestine measured after sacrifice for each treatment group. (**B**) Representative photographic images of the large intestine from mice in each group. (**C**) Immunohistochemical analysis of small intestine (SI) and colon (CO) tissues showing Lysozyme 1 and Mucin 2 expression, respectively (arrows indicate positive staining). Representative images are shown. (**D**) Quantification of Lysozyme 1-positive cells per field of view in small intestine tissue. (**E**) Quantification of Mucin 2-positive cells per field of view in colon tissue. No statistically significant differences were observed in large intestine length. Statistical significance for immunohistochemical analyses is indicated as * *p* = 0.0375 and **** *p* < 0.0001.

Macroscopic analysis of colon length in mice treated with postbiotics from *L. plantarum* and *B. mojavensis*, and with aguamiel, revealed no significant differences relative to the control group (Figure 2A,B), suggesting the absence of inflammation or macroscopic structural alterations.

Figure 2D,E shows lysozyme 1 expression in the small intestine and mucin 2 expression in the large intestine across all groups. The homogeneous basal expression indicates that the postbiotics did not affect key proteins involved in innate immunity or mucosal barrier integrity, with no evidence of histological damage. Quantitative data (Figure 2D) confirmed a similar number of lysozyme 1-positive cells among treatments. Mucin 2 expression, in contrast, was significantly increased in the *B. mojavensis* group relative to the control (Figure 2E), suggesting a potential strengthening of the intestinal barrier.

**Figure 3:**
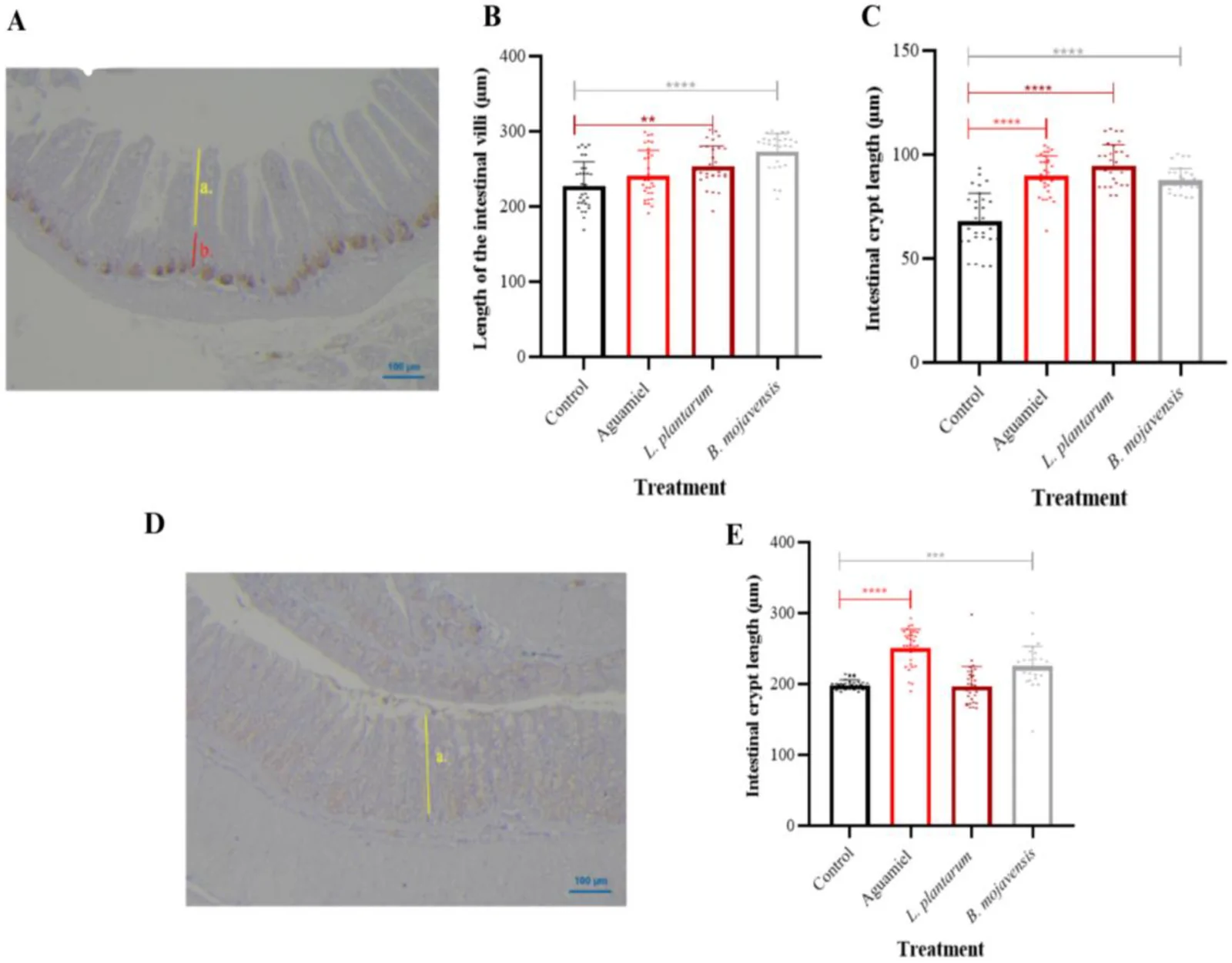
Effects of treatments on villus and crypt morphology in the small and large intestine of C57BL/6J mice. (**A**) Representative histological image of small intestine tissue showing the meas-urement of villus height (yellow line) and crypt depth (red line). Scale bar = 100 μm (a) villus and (b) crypt in the small intestine. (**B**) Villus length (µm) in the small intestine across treatment groups. (**C**) Crypt length (µm) in the small intestine across treatment groups. (**D**) Representative histological image in the large intestines tissue showing the measurement of crypt length (yellow line). Scale bar = 100 μm (a) intestinal crypt in the large intestines (**E**) Crypt length (µm) in the large intestine across treatment groups. Statistical significance is indicated as **** *p* < 0.0001, *** *p* = 0.0001, and ** *p* = 0.0017.

Histomorphometric analysis of the small intestine revealed significant elongation of both villi and intestinal crypts in animals treated with postbiotics from *L. plantarum* and *B. mojavensis*, relative to the control group (Figure 3B,C). Colon elongation, by contrast, was induced only by treatment with aguamiel and *B. mojavensis* (Figure 3D). Overall, these results indicate that postbiotics modify intestinal cytoarchitecture by promoting elongation of functional units, possibly through changes in intestinal mucosal homeostasis.

### 3.3. Powder Properties

**Figure 4:**
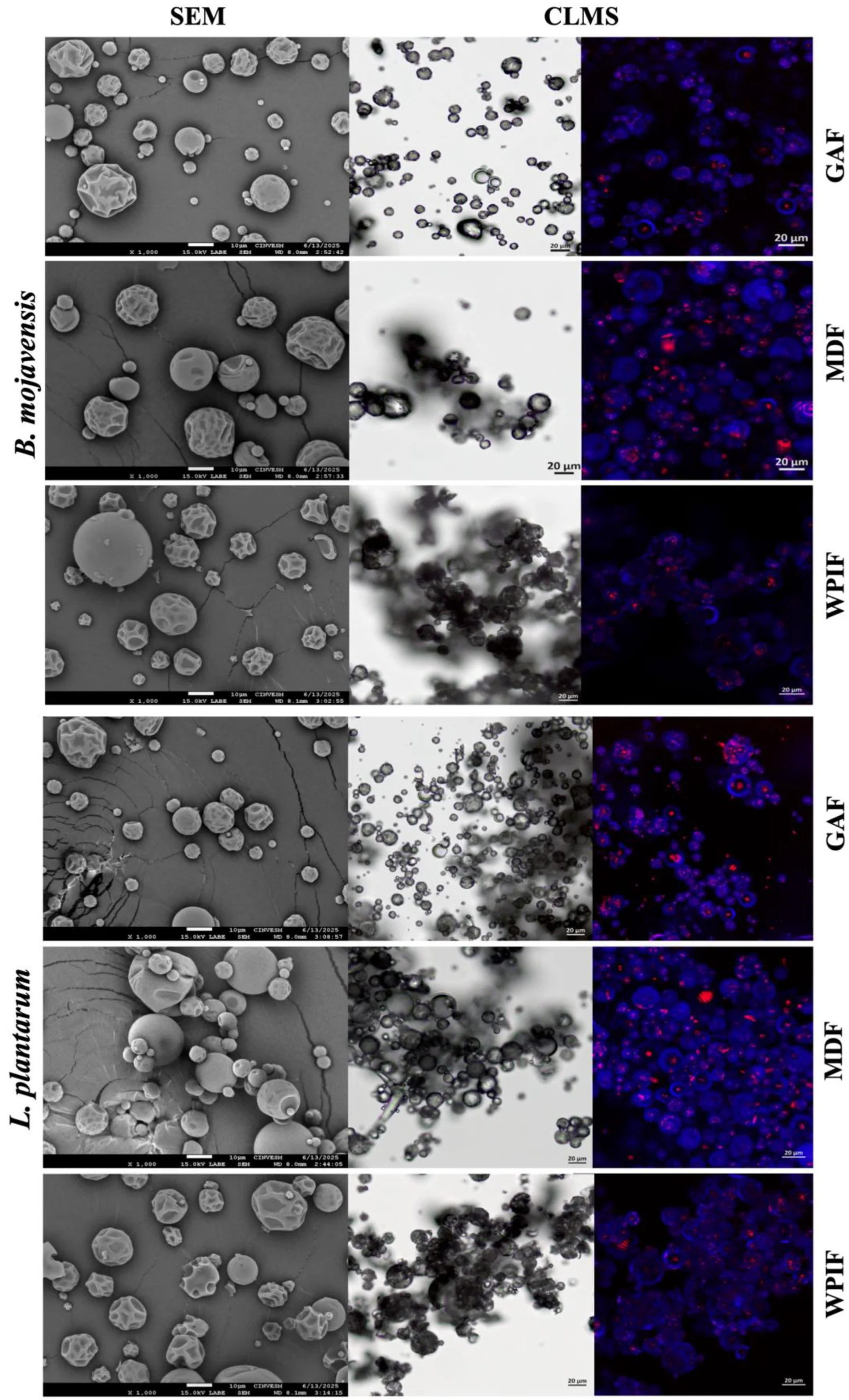
Microencapsulated postbiotics powders (SEM micrographs highlighting particle morphology and surface topology, CLSM images showing the microstructure of the encapsulating matrices and representative images of the powders).

**Figure 5:**
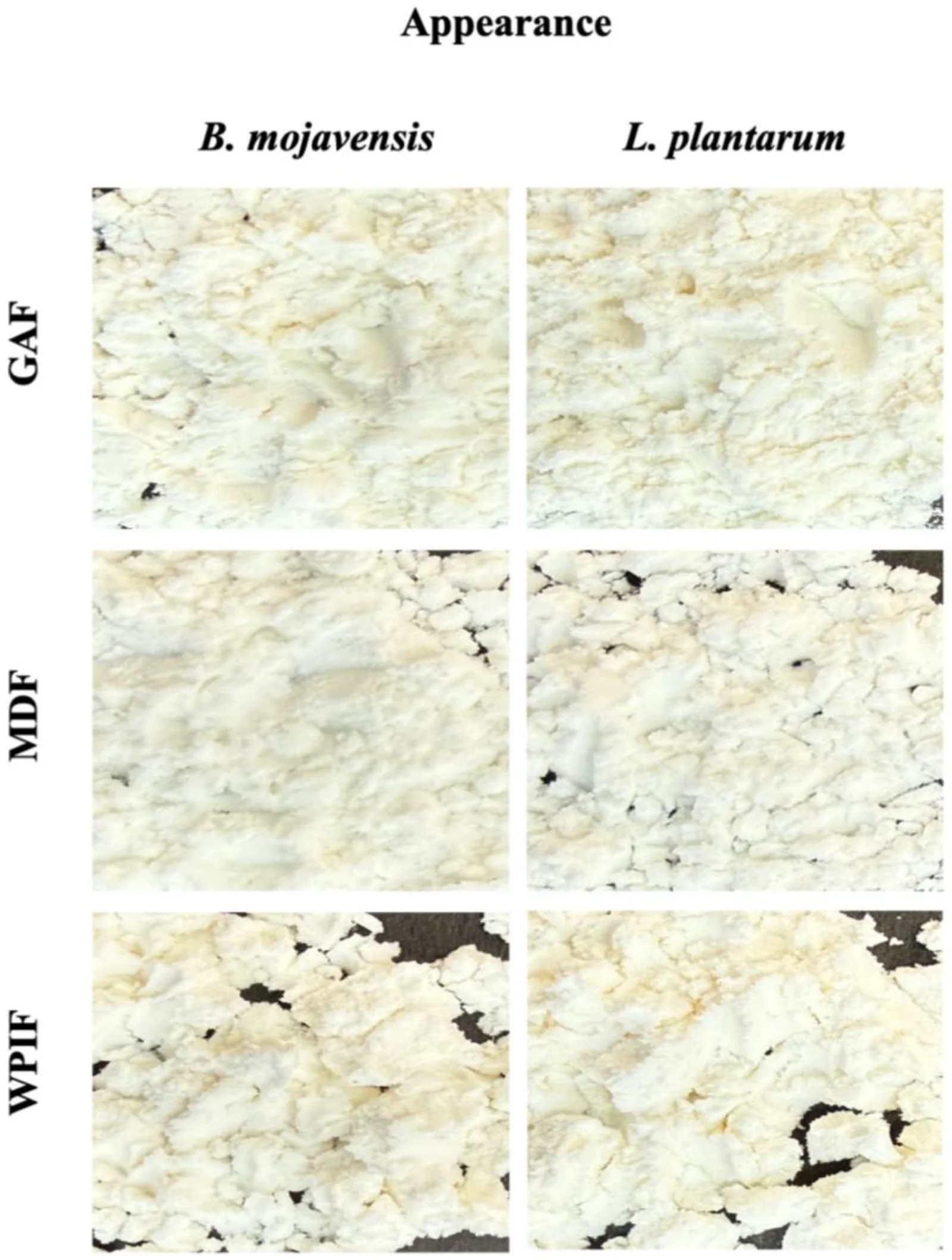
Macroscopic appearance of spray-dried postbiotic powders obtained from *B. mojavensis* and *L. plantarum* microfiltrates using different wall-material formulations (GAF, MDF, and WPIF).

The physicochemical properties of fermented aguamiel powders varied according to the microorganism used (*B. mojavensis* and *L. plantarum*) and the wall material employed (GAF, MDF, and WPIF). These results are presented in Table 2, Figure 4 and Figure 5 show the microscopic and macroscopic appearance, respectively, of the different treatments.

Moisture content and aw of the microcapsules from both microorganisms ranged from 3.55–6.38% and 0.11–0.17, respectively, with significant differences among treatments (*p* < 0.001; Table 2). Powder pH also differed significantly among treatments (*p* < 0.001): *L. plantarum* powders were more acidic (3.90–4.00), whereas *B. mojavensis* powders showed higher pH values (4.97–5.95). Color analysis likewise revealed significant differences (*p* < 0.001). MDF combined with *B. mojavensis* showed the highest lightness (61.33 ± 0.25), whereas GAF combined with *B. mojavensis* showed the lowest (46.47 ± 0.31). Parameter a* was negative in all treatments, indicating greenish tones, ranging from −1.60 to −0.73. The b* parameter ranged from 4.37 to 7.47, with the highest values in GAF combined with *L. plantarum*. Hue angle (h°) values ranged from 96.61 to 108.99.

Bulk density ranged from 0.23–0.50 g/mL for *B. mojavensis* and 0.25–0.48 g/mL for *L. plantarum*, with MDF treatments showing the highest values in both cases (*p* < 0.001; Table 3). This higher density may be associated with the formation of more compact, less porous particles, favored by the lower viscosity of MDF during spray drying. Tapped density followed the same trend, ranging from 0.42–0.68 g/mL. Hygroscopicity was significantly lower in MDF (10.73% for *B. mojavensis* and 9.74% for *L. plantarum*), whereas GAF showed the highest values (13.77% and 12.71%, respectively; *p* < 0.001). Process yield was highest for the MDF formulations, reaching 98.38% for *B. mojavensis* and 96.33% for *L. plantarum*; no significant differences were observed among treatments overall (*p* > 0.05), indicating low solid losses during spray drying.

Flowability indices (Carr index and Hausner ratio) confirmed significant differences among treatments. For *B. mojavensis*, WPIF exhibited very poor flowability (CI = 44%, HR = 1.80), whereas MDF showed more acceptable behavior (CI = 25%, HR = 1.34). For *L. plantarum*, the best flowability corresponded to GAF (CI = 15%, HR = 1.17), classified as good, indicating that gum arabic combined with fructans can generate less cohesive powders in this matrix. Solubility values were consistently high (≥83%), reaching 90.70% for GAF combined with *B. mojavensis* (*p* < 0.001); high solubility is a desirable attribute in encapsulation matrices, as it promotes rapid reconstitution in water. Wettability time varied widely among treatments and was shortest for MDF (41.72 s for *B. mojavensis* and 40.04 s for *L. plantarum*; *p* < 0.001), indicating rapid wetting and potential for instant reconstitution. WPIF, in contrast, showed markedly longer times (>150 s for *B. mojavensis*), which may relate to the partially hydrophobic nature of whey proteins, hindering initial water contact.

### 3.4. Microscopy

SEM analysis revealed the surface morphology of the microencapsulated powders obtained with the different wall materials (Figure 4 and Figure 5). MDF microcapsules containing *L. plantarum* and *B. mojavensis* showed relatively uniform, spherical particles with smoother surfaces and a lower degree of collapse than the other encapsulating materials. Microcapsules produced with GAF were likewise spherical but showed a greater tendency toward rough, partially collapsed surfaces in both strains. WPIF treatments, for both strains, presented heterogeneous particles with fractured surfaces and, in some cases, collapsed structures. The morphology observed by SEM correlated with the physicochemical properties reported above: MDF showed greater particle uniformity, consistent with its better flowability and lower hygroscopicity; the surface roughness of GAF is associated with its high solubility and water-absorption capacity; and the fractures observed in WPIF are consistent with its lower wettability and higher cohesiveness.

CLSM analysis enabled visualization of the internal distribution of the encapsulated components, clearly differentiating the wall matrix (blue fluorescence) from the active core (red fluorescence) corresponding to the microfiltrates of *B. mojavensis* and *L. plantarum* (Figure 4). Three-dimensional reconstruction (xyz axes) confirmed efficient, structurally intact encapsulation. GAF and WPIF treatments exhibited particles of heterogeneous size with irregular walls, whereas MDF produced more homogeneous microcapsules with an average size of approximately 20 µm.

### 3.5. FT-IR

**Figure 6:**
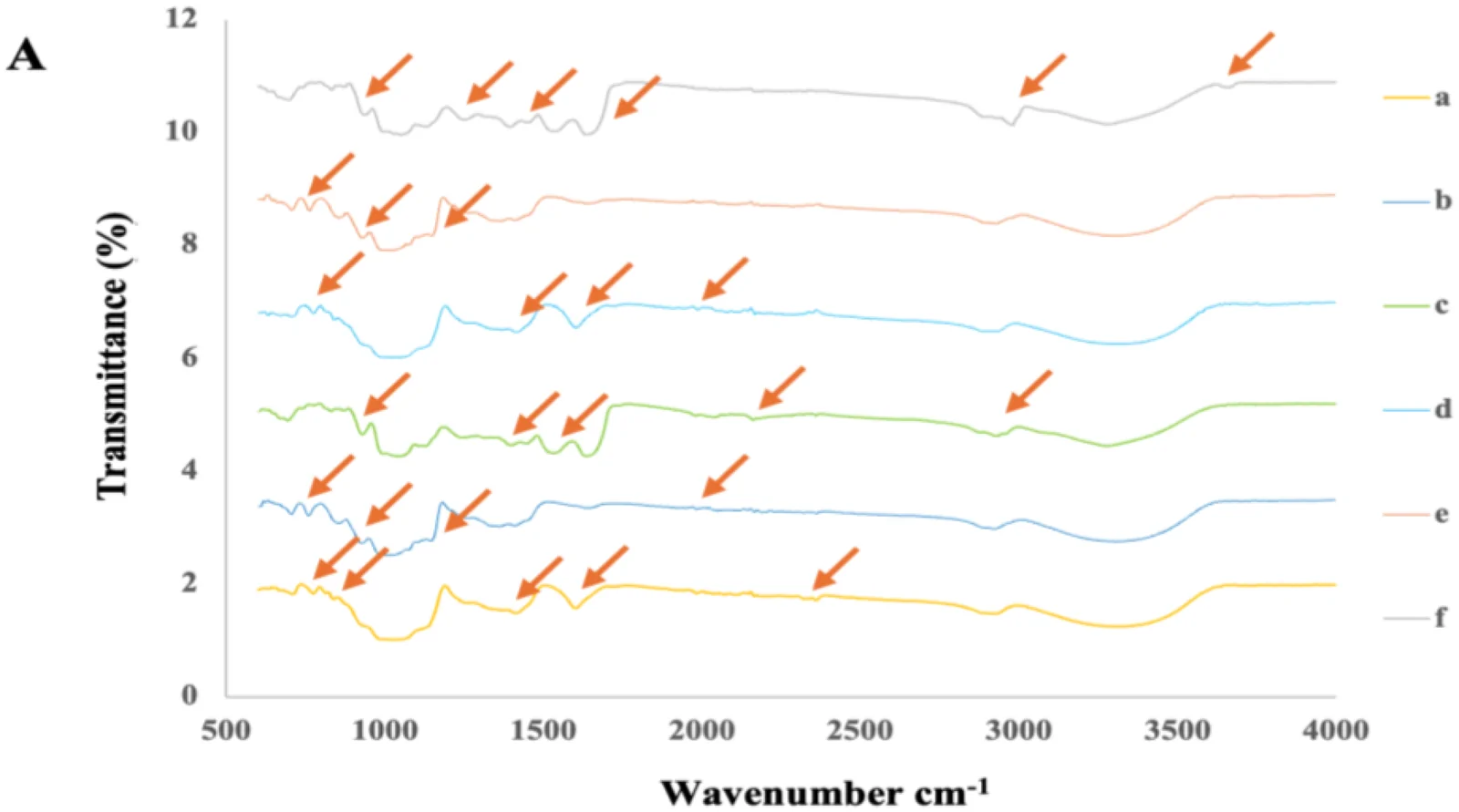

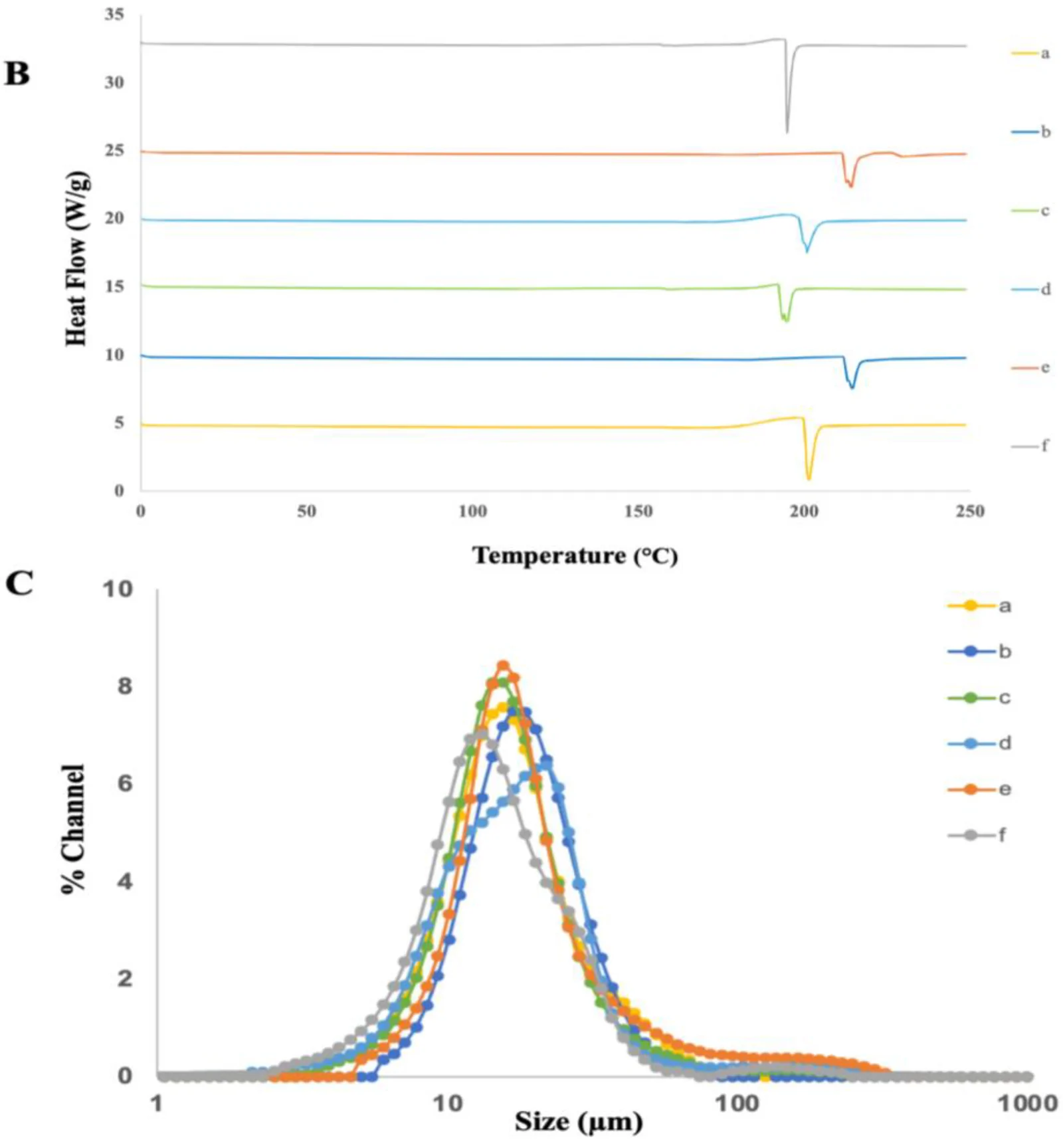
Molecular interactions, thermal behavior, and particle size distribution of spray-dried microencapsulated postbiotic systems. (**A**) FT-IR spectra showing predominant functional groups and potential protein–polysaccharide interactions. (**B**) DSC thermograms displaying thermal transitions, including Tg, and differences in thermal stability among encapsulating matrices. (**C**) particle size distribution of the resulting microcapsules. In all panels, (a–c) correspond to *B. mojavensis* with GAF, MDF, and WPIF, respectively, whereas (d–f) correspond to *L. plantarum* with GAF, MDF, and WPIF, respectively.

Figure 6A shows the FT-IR spectra of microcapsules obtained from treatments of fermented aguamiel with *L. plantarum* and *B. mojavensis*. A broad band was observed in the 3600–3000 cm^−1^ region, corresponding to –OH and –NH groups, with greater intensity in WPIF, indicating enhanced hydrogen bonding and bound water, together with the involvement of hydroxyl groups from polysaccharides and proteins. In the 2970–2850 cm^−1^ region, discrete peaks associated with aliphatic chain stretching vibrations (–CH_2_ and –CH_3_) were identified, related to lipids and particularly noticeable in MDF and WPIF. Bands in the 1743–1650 cm^−1^ region correspond to C=O stretching of triglycerides and amide I, while the signal near 1540 cm^−1^ corresponds to amide II (N–H bending), indicative of proteins and peptides. The 1450–1410 cm^−1^ region, particularly evident in GAF and MDF, is assigned to –CH_2_ and –CH_3_ deformation and carboxylate groups (–COO^−^), linked to fatty acids and carboxylate salts. The 1200–900 cm^−1^ region corresponds to the polysaccharide fingerprint (C–O–C and C–O), with intense peaks in GAF and MDF between 1045 and 1020 cm^−1^, attributable to fructans.

### 3.6. DSC

Figure 6B shows the thermograms for the different postbiotic treatments. A single, well-defined endothermic peak was observed in all samples. For treatments prepared with *B. mojavensis* postbiotics, melting temperatures of 200.81 °C (ΔH = 141.44 J/g) for GAF, 213.53 °C (ΔH = 100.07 J/g) for MDF, and 193.38 °C (ΔH = 94.82 J/g) for WPIF were obtained. Treatments with *L. plantarum* postbiotics showed melting temperatures of 202.02 °C (ΔH = 132.12 J/g) for GAF, 212.82 °C (ΔH = 99.87 J/g) for MDF, and 194.12 °C (ΔH = 116.40 J/g) for WPIF. These peaks are attributed to the melting of the wall materials used during microencapsulation. The largest temperature increase was observed for MDF in both microfiltrates, suggesting greater thermal stability of this system.

Regarding Tg, treatments containing *B. mojavensis* postbiotics showed values of 58.20 °C (GAF), 57.67 °C (MDF), and 51.20 °C (WPIF). For *L. plantarum* treatments, Tg values were 58.57 °C (GAF), 57.91 °C (MDF), and 51.48 °C (WPIF). In both cases, GAF exhibited the highest Tg values, suggesting greater resistance of the amorphous matrix to temperature-induced structural change.

### 3.7. Particle Size Characteristics

Figure 6C shows the particle size distribution of the powders obtained under the different treatments. MDF produced particles with a monomodal, narrower, and more homogeneous distribution, with an average size close to 17.64 µm; this distribution may favor technofunctional properties such as flowability, solubility, and storage stability. GAF and WPIF treatments, in contrast, exhibited broader distributions with a higher proportion of large particles, potentially associated with increased cohesiveness, lower dispersibility, and limitations during rehydration or mixing in *L. plantarum* formulations.

For *B. mojavensis*, all three treatments showed relatively normal distributions centered within the 16.15–18.51 µm range at their peaks, although differences were observed. MDF again showed a monomodal, narrower, more homogeneous distribution, representing an advantage for industrial applications requiring reproducibility and flow control. WPIF reached peak values comparable to MDF but showed greater dispersion toward larger particle sizes, which may adversely affect flowability and increase agglomerate formation. GAF showed a distribution slightly shifted toward larger sizes with a more extended tail, indicating lower uniformity.

Overall, these results indicate that MDF is the most efficient treatment for producing controlled homogeneous particles, whereas GAF and WPIF show a greater tendency toward heterogeneous fractions and agglomeration. Table 4 presents a comparison of the characteristic diameters (*D*_90_, *D*_50_, and *D*_10_) of the microencapsulated powders obtained in this study; the SPAN parameter describes the width of the particle size distribution.

## 4. Discussion

### 4.1. Phase 1: Biological Assays

#### 4.1.1. Antiproliferative Activity of Postbiotics of *L. plantarum* and *B. mojavensis* on Breast Cancer Cells

The antiproliferative effect of cell-free supernatants has been attributed to metabolites such as organic acids, bacteriocins, exopolysaccharides, and antioxidant compounds with cytotoxic activity, particularly conjugated linoleic acid (CLA), which is known to inhibit cell proliferation and induce selective apoptosis in malignant cells [30,31]. CLA has additionally been reported to promote cell-cycle arrest and enhance the efficacy of chemotherapeutic agents such as tamoxifen [32]. Given that dietary carcinogens can induce uncontrolled proliferation and DNA damage [33], postbiotics represent a promising alternative with chemopreventive potential. Cell-free supernatants derived from *Lactobacillus rhamnosus* have been shown to induce selective apoptosis through both intrinsic and extrinsic pathways [34,35]. Aguamiel, rich in fructooligosaccharides (FOS) and native microbiota including *L. plantarum* and *B. mojavensis*, thus represents a relevant source of postbiotic-producing strains with considerable biotechnological and therapeutic potential [18].

In the present study, postbiotics from *L. plantarum* and *B. mojavensis* significantly inhibited proliferation of MDA-MB-231 cells, in agreement with reports describing apoptosis regulation and cell-cycle modulation by lactobacilli metabolites [31,32]. No previous reports address *B. mojavensis* in the context of breast cancer; however, a reduction greater than 75% in SW-480 cell viability, without affecting healthy PBMCs, has previously been documented [35], supporting the selectivity of this strain’s metabolites.

#### 4.1.2. Effects of Postbiotics of *L. plantarum* y *B. mojavensis* on Intestinal Architecture and Mucosal Barrier Integrity

In the murine model, the transient reduction in body weight observed in our preliminary findings was consistent with previous reports and appears to lack clinical relevance [36]. Colon shortening is considered a classical indicator of inflammation and tissue damage in experimental colitis models [37,38]; the preserved colon length observed here therefore suggests the absence of inflammation and indicates that *ad libitum* administration of postbiotics for eight days did not compromise intestinal integrity, consistent with studies reporting the safety and lack of toxicity of lactobacilli-derived postbiotics in murine models [10,39].

Histologically, lysozyme 1 expression was confirmed in the small intestine, consistent with the antimicrobial function of Paneth cells. Mucin 2 was expressed in all groups, with a significant increase in the *B. mojavensis* group, suggesting strengthening of the mucosal barrier—an effect previously described for postbiotics derived from bacilli and lactobacilli capable of stimulating protective mechanisms against pathogens [10,39].

Histomorphometric analysis revealed preserved intestinal architecture, with significant elongation of villi and crypts, particularly in the *B. mojavensis* group. These changes are associated with increased absorptive capacity and epithelial renewal and are consistent with effects previously described for lactobacilli- and bacilli-derived metabolites in murine models [10,23,40,41]. Since villus atrophy is linked to malabsorption and intestinal dysfunction [42], these findings support the functional potential of the postbiotics evaluated here. Overall, postbiotics derived from aguamiel—particularly those produced by *L. plantarum* and *B. mojavensis*—exhibit cytotoxic activity together with favorable modulation of intestinal homeostasis, without inducing inflammation, positioning them as promising candidates for nutraceutical and therapeutic applications.

### 4.2. Phase 2: Properties and Characteristics

#### 4.2.1. Powder Characteristics

Gum arabic–fructans (GAF) treatments generally exhibited the highest moisture and/or a_w_ values, likely attributable to the high density of hydroxyl groups in the branched arabinogalactan structure, which favors water adsorption [43,44]. Maltodextrin–fructans (MDF), in contrast, showed the lowest moisture and a_w_ values, consistent with its role as a wall material that preserves the glassy structure and depresses a_w_ [45].

In whey protein isolate–fructans (WPIF) formulations, *B. mojavensis* samples exhibited low moisture and a_w_, attributable to formation of a continuous surface film that limits water exchange, as protein structural changes and intermolecular interactions reduce water mobility [46]. *L. plantarum* microcapsules, in contrast, presented higher a_w_ despite intermediate moisture content, explained by plasticization of the protein matrix caused by organic acids that lower the glass transition temperature and increase the free-water fraction [47]. These data are consistent with values reported by Jiménez-Sánchez et al. (2017) [48] and García-Solís et al. (2022) [28] (≈5% moisture and 0.17 a_w_). The stabilizing effect of maltodextrin, owing to its high Tg, has similarly been shown to reduce aw in spray-dried juices [49,50]; Tontul and Topuz (2017) [51] indicate that powders with aw below 0.3 are considered microbiologically and chemically stable, since such conditions limit microbial growth and oxidative degradation.

The observed pH differences reflect the microbial metabolism of the two organisms: *L. plantarum* produces organic acids (mainly lactic and acetic), lowering pH [52], whereas Bacillus spp. are reported to synthesize metabolites such as peptides and lipopeptides that are less acidic [53]. Final pH is further influenced by the wall material—protein-based matrices can buffer pH changes through ionic interactions with acids, whereas polysaccharides such as gum arabic exhibit lower buffering capacity [54].

The color results are explained by Maillard reactions and caramelization: whey protein isolate participates actively in browning reactions, darkening the samples and increasing b*, whereas maltodextrin, lacking amino groups, yields lighter powders [55]. Gum arabic, in contrast, can retain natural pigments, intensifying the yellow color [27], and Goula and Adamopoulos (2008) [49] similarly demonstrated that maltodextrin produces lighter, more stable colors. Ortiz-Basurto et al. (2017) [24], using three wall materials including a combination of maltodextrin and agave fructans, likewise reported no significant differences in color.

In agreement with Tontul and Topuz (2017) [51], who reported that matrices with higher solid content produce denser, less aerated particles, our results indicate the formation of more compact, less porous structures—an effect associated with the high matrix-forming capacity of maltodextrin as a carrier [52]. Fernandes et al. (2014) [54], using a gum arabic–inulin mixture as wall material, reported that higher density facilitates industrial handling, improves powder flowability, and reduces packaging volume. These results confirm that maltodextrin, owing to its high Tg, reduces moisture uptake and the risk of agglomeration [49], whereas gum arabic, owing to its highly hydrophilic nature, promotes water absorption [56], supporting the advantage of MDF over GAF or WPIF in reducing hygroscopicity. The literature further indicates that maltodextrin incorporation in sugar-rich juices increases yield by reducing stickiness, preventing deposition in the drying chamber, and minimizing wall adhesion [55,57]; Mohammed et al. (2020) [58] likewise reported that materials with higher Tg, such as maltodextrin, improve drying efficiency relative to proteins or gums. Other studies have reported encapsulation efficiencies of up to 95% in combined protein–polysaccharide matrices [15].

According to Fitzpatrick et al. (2004) [59], CI values > 30% and HR values > 1.45 are associated with high cohesiveness, consistent with the results observed for WPIF and MDF in *L. plantarum*. Bae and Lee (2008) [60] similarly highlighted that wall material type determines the flowability of microencapsulated powders, consistent with the present findings. These results also agree with Mohammed et al. (2020) [58], who reported that maltodextrin improves powder dispersion and dissolution owing to its hydrophilic character, and with Sarabandi et al. (2018) [56], who observed that gum arabic and WPC increase hydration capacity and dispersibility relative to maltodextrin. Similarly, Bustamante et al. (2020) [61] observed that proteins tend to delay initial wetting through formation of a less permeable surface layer. Overall, the powders obtained showed high solubility (>85%) and rapid wettability (<1 min) in several treatments—desirable characteristics for instant-beverage applications, consistent with values reported in other studies on probiotic microencapsulation [16] and plant-extract encapsulation [27].

#### 4.2.2. Morphological Characteristics

Particles were predominantly spherical, although surface collapse, fracturing, and roughness were also observed, indicating that the wall material strongly influences the final microcapsule structure [62,63]. This behavior is associated with the high Tg of maltodextrin and its ability to form continuous films that stabilize droplets during spray drying, yielding powders with lower hygroscopicity and improved flowability [62]. In GAF systems, surfaces appeared more irregular, with some agglomeration, consistent with the heteropolysaccharide nature of gum arabic and its limited capacity to form continuous matrices [63]; these findings agree with the high hygroscopicity of gum arabic, since moisture absorption promotes deformation of the surface structure [62,64]. The morphology observed for WPIF is attributable to protein denaturation during drying and the lower mechanical resistance of protein-based matrices relative to polysaccharide systems [65]; overall, WPIF treatments showed a greater degree of agglomeration and surface roughness, consistent with their lower flowability and reduced wettability [63]. These findings indicate that the capacity of each material to form stable films, resist rapid evaporation, and maintain interfacial properties determines the final particle morphology [62,64].

CLSM is a valuable tool for distinguishing multiple components non-destructively [28,66]; recent studies have similarly shown that matrices combining fructans or inulin with maltodextrin or gum arabic provide more defined, uniform internal distributions, improving structural visualization by techniques such as CLSM and SEM [64].

#### 4.2.3. Infrared Spectral Analysis

In agreement with Barzola et al. (2025) [67], and consistent with other reports, microcapsules containing green coffee extract have shown a 94% spectral correlation with wall materials (fructans and gum arabic), suggesting interaction between the wall material and the encapsulated components [28]. Juárez-Trujillo et al. (2024) [15] observed that microcapsules produced with WPC and fructans showed defined peaks at 2922 and 2853 cm^−1^, as well as a band at 1743 cm^−1^, evidencing the formation of protein–polysaccharide complexes that confer greater thermal and structural stability; this observation agrees with Cerero-Calvo et al. (2022) [68], who reported that fermentation metabolites (lactate, acetate, succinate) contribute to the presence of these functional groups, and with Vázquez-Vuelvas et al. (2020) [69], characteristic of inulin, cellulose, and agavins present in fructans. This result is further consistent with García-Solís et al. (2022) [28], who demonstrated that fructans–gum arabic combinations generate matrices with Tg ≈ 80 °C, improving thermal stability and reducing hygroscopicity.

#### 4.2.4. Glass Transition Temperature

These data agree with Rosland Abel et al. (2020) [70], who evaluated gum arabic and reported Tg values between 56.18 and 57.62 °C. A higher glass transition temperature is associated with reduced molecular mobility in the amorphous matrix, which may contribute to improved physical stability and greater resistance to thermal and structural change; accordingly, Tg was interpreted here as an indicator of amorphous-matrix stability rather than a direct measure of crystallinity. Siemons et al. (2020) [71] similarly reported that low-dextrose-equivalent maltodextrin (≤12) presents a broader molecular weight distribution and higher Tg than high-dextrose-equivalent maltodextrin (≥21), favoring performance in microencapsulation processes. Gullifa et al. (2023) [5] emphasized the relevance of selecting wall materials with appropriate Tg, as this enables higher yields during spray drying.

#### 4.2.5. Particle Size Distribution

These findings demonstrate that the wall material has a significant impact on particle size distribution. WPIF was the most efficient at producing smaller microcapsules with a narrower size distribution, which may influence powder handling and reconstitution properties in aqueous media—parameters relevant to applications in functional food matrices [72]. Similar results were reported by García-Solís et al. (2022) [28] in the optimization of gum arabic–fructans mixtures. The effect of temperature and carrier on powder flow properties is likewise consistent with the findings of Barradas-Pretelín et al. (2022) [73], who evaluated spray-dried avocado powders using maltodextrin as the wall material.

D_50_ values obtained are consistent with those reported by Aita et al. (2022) [74], who obtained a D_50_ of 15.82 µm using maltodextrin as wall material. Similar results were reported by Lourenço et al. (2020) [75], who used inulin and pineapple peel extract and obtained size distributions ranging from 1.3 to 18.2 µm, with average diameters below 12 µm—slightly lower than those found in the present study, suggesting that drying conditions and feed composition strongly modulate the resulting microstructure. Jiménez-Sánchez et al. (2017) [48] attributed such differences to the presence of β-(2→6) linkages in native agave fructans, which confer greater flexibility to the biopolymer chains, reduce Tg, and may promote particle agglomeration during spray drying; in agreement, García-Solís et al. (2022) [28] indicated that the agglomeration observed in fructans-based treatments relates to their high water-absorption capacity, arising from the abundance of hydroxyl groups that facilitate interaction with water vapor during drying. Additional studies highlight that combinations of gum arabic, inulin, and proteins as wall materials generate microcapsules with heterogeneous morphologies and irregular surfaces, influenced by core composition, droplet size, and spray-drying conditions [72,75]. Controlling particle size is important for powder stability and flow properties [26], as well as for compressibility and dispersibility during reconstitution [71]; the SPAN parameter describes the width, or heterogeneity, of the particle size distribution [51,76,77].

### 4.3. Study Limitations and Future Perspectives

This study was designed as a characterization and safety-evaluation stage, a fundamental step in the development of new postbiotic ingredients prior to their evaluation in disease models. Administration of the postbiotics to healthy mice not only confirmed the absence of intestinal damage but also revealed favorable modulatory effects on intestinal architecture and barrier function (villus and crypt elongation, increased mucin 2), providing novel evidence of the biofunctional potential of these native aguamiel strains. The sample size (*n* = 3 per group), although consistent with exploratory studies, limits statistical power and should be confirmed in a larger cohort. Similarly, antiproliferative activity was assessed using the MTT assay, a widely validated initial screening method; complementary assays (LDH release, flow cytometry) will provide further insight into the mechanisms underlying the observed effect. Our group is currently conducting studies in murine models of induced intestinal damage to directly evaluate the anti-inflammatory and protective effects of these postbiotics, building on the findings reported here. Additionally, histological processing of the intestinal tissue (embedding, sectioning, and staining) was performed by a specialized external service, so complete technical protocol details are not available, which may limit exact reproducibility. Histomorphometric measurements of villi and crypts were performed by a single evaluator aware of the experimental group identity, without blinding, which could introduce selection bias; future studies should incorporate blinded measurements by independent evaluators.

This research provides relevant findings in the field of functional foods, particularly regarding powdered postbiotics obtained from aguamiel, a traditional Mexican beverage substrate, supported by initial biological validation. Metabolomic analyses of these postbiotics are currently underway to identify the specific metabolites responsible for the observed biological effects; this characterization will further enable determination of encapsulation efficiency and loading capacity. The present study thus establishes a solid foundation for future research on this postbiotic ingredient, including shelf-life evaluation, preclinical intervention studies, technology transfer to aguamiel-producing communities, targeted metabolomic analyses, and other efforts aimed at supporting its development as a functional food ingredient.

## 5. Conclusions

Postbiotics derived from *L. plantarum* and *B. mojavensis* fermented in aguamiel demonstrated significant biological activity and technological feasibility for the development of functional ingredients. Both postbiotics significantly reduced cell viability, indicating cytotoxic activity against MDA-MB-231 cells, without inducing macroscopic or histological intestinal alterations under the conditions evaluated (oral administration for eight days in healthy mice), suggesting the absence of short-term intestinal damage. Additional studies incorporating biochemical and hematological panels, and of longer duration, are needed to establish a broader nutraceutical safety profile.

These results demonstrate that postbiotics derived from fermented aguamiel constitute a promising source of bioactive compounds, and that spray drying represents an appropriate processing strategy, yielding powders with favorable technological properties for potential incorporation into food matrices. Further studies are nonetheless required to identify the metabolites responsible for the observed biological effects, evaluate encapsulation efficiency and storage stability, and confirm the efficacy of the microencapsulated postbiotics in preclinical and clinical models before health-related applications can be considered.

## Figures and Tables

**Table 1:** Treatments of postbiotics with encapsulating material.

Bacteria	Treatment
*B. mojavensis*	Gum Arabic + Fructans (GAF)
	Maltodextrin + Fructans (MDF)
	Whey Protein Isolate + Fructans (WPIF)
*L. plantarum*	Gum Arabic + Fructans (GAF)
	Maltodextrin + Fructans (MDF)
	Whey Protein Isolate + Fructans (WPIF)

**Table 2:** Physicochemical parameters of the microencapsulated postbiotics powders.

	*B. mojavensis*			*L. plantarum*			
	GAF	MDF	WPIF	GAF	MDF	WPIF	*p*-Value
a_w_	0.16 ± 0.01 ^ab^	0.14 ± 0.01 ^b^	0.11 ± 0.03 ^ab^	0.17 ± 0.00 ^a^	0.11± 0.00 ^c^	0.17 ± 0.00 ^c^	*p* < 0.001
Moisture content (%)	6.38 ± 0.14 ^a^	5.98 ± 0.12 ^ab^	5.43 ± 0.27 ^b^	3.94 ± 0.26 ^cd^	3.55 ± 0.25 ^d^	4.47 ± 0.33 ^c^	*p* < 0.001
pH	4.97 ± 0.06 ^c^	5.53 ± 0.06 ^b^	5.95 ± 0.06 ^a^	4.00 ± 0.00 ^d^	3.90 ± 0.00 ^c^	5.47 ± 0.06 ^b^	*p* < 0.001
L*	46.47 ± 0.31 ^d^	61.33 ± 0.25 ^a^	55.67 ± 0.40 ^c^	56.40 ± 0.36 ^c^	59.87 ± 0.12 ^b^	60.63 ± 0.29 ^ab^	*p* < 0.001
a*	−1.6 ± 0.26 ^c^	−1.33± 0.15 ^bc^	−1.10 ± 0.20 ^abc^	−0.87 ± 0.12 ^ab^	−1.50 ± 0.10 ^c^	−0.73 ± 0.10 ^a^	*p* < 0.001
b*	6.37 ± 0.21 ^b^	4.37 ± 0.21 ^c^	5.90 ± 0.17 ^b^	7.47 ± 0.15 ^a^	4.37 ± 0.15 ^c^	6.23 ± 0.23 ^b^	*p* < 0.001
h°	104.12 ± 1.44 ^b^	106.99 ± 0.95 ^a^	100.59 ± 1.12 ^b^	96.61 ± 0.77 ^c^	108.99 ± 0.79 ^a^	96.75 ± 1.46 ^c^	*p* < 0.001

Results represent the mean ± standard deviation (*n* = 3). ^a–d^ Means with different lowercase superscript letters within the same row indicate statistically significant differences (*p* < 0.05). GAF: Gum arabic + Fructans, MDF: Maltodextrin + Fructans and WPIF: Whey Protein Isolate + Fructans.

**Table 3:** Physical properties of postbiotics microencapsulates.

Propiedades	*B. mojavensis*			*L. plantarum*			
	GAF	MDF	WPIF	GAF	MDF	WPIF	*p*-Value
Bulk density (g/mL)	0.48 ± 0.02 ^a^	0.50 ± 0.00 ^b^	0.23 ± 0.01 ^c^	0.48 ± 0.02 ^a^	0.39 ± 0.01 ^b^	0.25 ± 0.01 ^c^	*p* < 0.001
Tapped density (g/mL)	0.68 ± 0.02 ^a^	0.67 ± 0.01 ^a^	0.42 ± 0.01 ^c^	0.56 ± 0.02 ^b^	0.57 ± 0.02 ^b^	0.36 ± 0.01 ^d^	*p* < 0.001
Higroscopicity (%)	13.77 ± 0.54 ^a^	10.73 ± 0.25 ^c^	11.37 ± 0.10 ^b^	12.71 ± 0.56 ^a^	9.74 ± 0.37 ^d^	11.46 ± 0.21 ^b^	*p* < 0.001
Process yield (%)	93.24 ± 2.12 ^a^	98.38 ± 0.73 ^a^	96.87 ± 1.04 ^a^	93.12 ± 2.52 ^a^	96.33 ± 0.66 ^a^	93.08 ± 1.96 ^a^	*p* > 0.066
Carr Index	0.29 ± 0.03 ^b^	0.25 ± 0.01 ^c^	0.44 ± 0.03 ^a^	0.15 ± 0.02 ^d^	0.32 ± 0.03 ^b^	0.32 ± 0.02 ^b^	*p* < 0.001
Hausner Index	1.40 ± 0.05 ^bc^	1.34 ± 0.02 ^c^	1.80 ± 0.10 ^a^	1.17 ± 0.03 ^d^	1.48 ± 0.07 ^b^	1.47 ± 0.04 ^bc^	*p* < 0.001
Solubility (%)	90.70 ± 0.66 ^a^	83.87 ± 0.21 ^d^	89.20 ± 0.35 ^b^	84.03 ± 0.35 ^d^	84.30 ± 0.35 ^d^	87.87 ± 0.35 ^c^	*p* < 0.001
Wettability (s)	134.05 ± 2.79 ^b^	41.72 ± 1.50 ^e^	157.53 ± 3.50 ^a^	86.08 ± 3.59 ^d^	40.04 ± 2.16 ^c^	100.40 ± 0.60 ^c^	*p* < 0.001

Results represent the mean ± standard deviation (*n* = 3). ^a–e^ Means with different lowercase superscript letters within the same row indicate statistically significant differences (*p* < 0.05). GAF: Gum arabic + Fructans, MDF: Maltodextrin + Fructans and WPIF: Whey Protein Isolate + Fructans.

**Table 4:** Characteristic parameters of particle size distribution.

	Treatments	*D*_10_	*D*_50_	*D*_90_	SPAN
*L. plantarum*	GAF	8.29	17.49	33.33	1.43
	MDF	10.32	17.64	48.35	2.16
	WPIF	7.52	14.52	30.40	1.58
*B. mojavensis*	GAF	8.97	16.46	34.30	1.54
	MDF	11.27	18.51	49.56	2.07
	WPIF	9.12	16.15	31.57	1.39

GAF: Gum arabic + Fructans. MDF: Maltodextrin + Fructans. WPIF: Whey Protein Isolate + Fructans.

## Data Availability

The original contributions presented in this study are included in the article. Further inquiries can be directed to the corresponding author.
